# A new water-based topical carrier with polar skin-lipids

**DOI:** 10.1186/1476-511X-5-12

**Published:** 2006-05-03

**Authors:** Mats Silvander, Lovisa Ringstad, Ruby Ghadially, Thomas Sköld

**Affiliations:** 1 YKI, Institute for Surface Chemistry, Box 5607, SE-114 86 Stockholm, Sweden; 2 Department of Pharmacy, Uppsala University, Box 580 SE-751 23, Sweden; 3 University of California, VA Medical Centre (190), 4150 Clement St., San Francisco, CA94118, USA; 4 Björnö Gård, SE-761 41 Norrtälje, Sweden

## Abstract

A new water-based topical formulation is presented that aims at providing good penetration properties for both lipophilic and hydrophilic drugs with as small a disturbance of the skin barrier function as possible. The formulation contains dispersed lipids in a ratio resembling that of human skin. The capacity to deliver is addressed in this first study while the mild effect on skin will be presented later. Three variations of the lipid formulation were investigated by use of pigskin *in vitro *diffusion cell. The hydrophilic 5(6)-carboxyfluorescein (CF) and the lipophilic acridine orange 10-nonyl bromide (AO) were used as model drug substances. The results showed that the delivery properties of the new formulation exceeded that of the references (vaseline and xanthan gum gel). The effect was largest for lipophilic AO where all lipid matrix formulations were superior in amount detected in the skin. The results for the hydrophilic CF were also promising. Especially efficient was the lipid formulation containing the non-ionic adjuvants tetra ethylene glycol monododecyl ether and polyoxyethylene 23 dodecyl ether. The additional *in vivo *study suggests that the used *in vitro *model has qualitative bearing on relevant *in vivo *situations.

## Introduction

The human skin, and especially epidermis, constitutes an efficient barrier for foreign substances to penetrate the skin. The ability to circumvent this barrier depends on the properties of the foreign substance as well as the status of different parts of the skin. For instance it has been reported that lipophilic small molecules in general penetrate deeper than do larger hydrophilic ones [1,2]. The thickness of the epidermis varies but is in the order of a few hundred μm and consists of stratum corneum, stratum granulosum, stratum spinosum and the stratum basale. The stratum corneum gives the main contribution to the barrier function against diffusion across the skin [3]. The stratum corneum consists of corneocytes surrounded by lipids and is commonly described by the brick-and-mortar model [4,5]. Stratum corneum lipids in general are long-chained and have high chain-melting temperatures [6]. For the purpose of drug delivery via topical administration it is required that a large enough amount of drug should successfully penetrate the SC and reach the target in order to obtain therapeutic effects. In order to facilitate penetration so called enhancers are often used together with the drug. Several enhancers, such as ethanol, dimethylsulfoxide and terpenes, have been shown successful in their task to promote penetration [7-9]. A common side effect of these enhancers is on the other hand that the barrier function of SC is disturbed for long periods of time after skin application [7,10]. This results in a more sensitive skin and irritancy. In addition, if the disturbance of the barrier function has a long time span the risk of unwanted uptake increases.

The choice of formulation for the drug is also important in order to obtain a suitable release profile, solubility/dispersability and stability of the drug. There are numerous examples where a suitable carrier system dramatically improves drug efficacy without the effect being solely penetration enhancement as such [3]. The most functional formulation should therefore be able to solubilise both hydrophilic and lipophilic substances and at the same time increase uptake efficiently without causing notable damage to the skin. In this context, a prerequisite for the present study was that several studies showed that the presence of lipid materials that resembles the ratio of human skin are beneficial for barrier recovery [10-13]. The present study contains a topical formulation that consists of dispersed lipids with a composition that resembles that of human stratum corneum. The lipid part consists of fatty acids, cholesterol, phospholipids and ceramide. The phospholipids are a substitute for a large part of the ceramides due to commercial reasons. The formulation is formed by mixing three different fractions that are processed individually. A mixer is used to form a disperse lipid phase and a sonicator to form an ultra-fine disperse lipid phase. The latter process results in vesicles, a type of carrier that is frequently studied in dermatology and has been shown to increase the delivered amount of drug both for lipophilic and hydrophilic drugs [3,6,14]. The two dispersed lipid phases are mixed together and a third phase, the polymer phase, that contributes to viscosity and texture is added. Consumer compliance has been important for the development of the formulation and the idea is to combine small submicron vesicles beneficial for delivery with larger dispersed structures that contributes to the lubricating feeling on skin. In the present study the focus is on penetration properties of the formulation.

The *in vitro *penetration on pig-skin of three different versions of the formulation were investigated and compared with appropriate references. Both hydrophilic and hydrophobic model actives were used. Any evidence of a rapid repair of the barrier function is the focus of a coming study and will not specifically be addressed here.

## Materials and methods

### Materials

Epikuron 200SH was obtained from Degussa BioActives (Hamburg, Germany). Cholesterol, triethanol amine and phenonip (preservative) were obtained from Vendico Chemical (Malmö, Sweden). SK-influx was obtained from Goldschmidt Personal Care, Essen, Germany. Mevalonic acid, propylene glycol, glycerol, polyvinylpyrrolidone, xanthan gum, tetraethylene glycol monododecyl ether, polyoxyethylene 23 dodecyl ether and 5(6)-carboxyfluorescein (CF) were obtained from Sigma. HEPES was obtained from Acros Organics. Acridine orange 10-nonyl bromide (AO) was obtained from Fluka. Sodium hydroxide and sodium chloride were obtained from Merck. Vaseline (petrolatum gel) was purchased from Apoteksbolaget AB, Stockholm, Sweden. The water used was double distilled, deionised and filtered with a Milli-Q system.

### Manufacturing

Typically ten grams of total formulation was prepared containing: 7.95 g water, 0.35 g Epikuron 200SH, 0.15 g cholesterol, 0.15 g palmitic acid, 0.2 g SK-influx, 0.4 g propylene glycol, 0.3 g glycerol, 0.2 g polyvinyl pyrrolidone, 0.2 g xanthan gum, 0.05 g triethanol amine, 0.04 g Phenonip, 0.01 g mevalonic acid. The pH was adjusted to 7.4–7.6 by use of NaOH. The concentrations used for the model drugs were 60 mM for the lipophilic AO and 30 mM for the hydrophilic CF. In case any other additives were added (e.g. adjuvants) the amount of water was reduced so that all other components were kept at the level described above.

#### Dispersed lipid phase

Half of the lipid material (including cholesterol) was added to a third of the total water. Half of the triethanol amine, half of the phenonip and half of the model active were also added unless otherwise stated. The fraction was heated to 70°C whilst stirring for approximately 30 minutes. The fraction was processed with an Ultra Turrax homogeniser for one minute at a speed of 9500 rpm. After this the fraction was let to cool down. The process results in a homogeneous lipid dispersion.

#### Ultra-fine dispersed lipid phase

The ultra-fine dispersed phase has the same chemical composition as the disperse lipid phase. This fraction was also heated to 70°C whilst stirring for approximately 30 minutes. The fraction was sonicated by use of a Soniprep 150, (Sanyo Gallencamp Plc) for 30 minutes. After this the fraction was let to cool down. The process creates a vesicle dispersion.

#### Polymer phase

The propylene glycol, glycerol, polyvinyl pyrrolidone and xanthan gum was added to a third of the total water. The fraction was heated to 70°C whilst stirring for approximately 30 minutes after which the fraction was left to cool down.

#### Mixing

The three phases were added in a beaker and the mix was stirred manually for 5 minutes.

#### Reference formulations

The xanthan gum gel was manufactured by heating the appropriate amount of water to 70°C and adding thereafter adding 2% xanthan gum. The gel was stirred until no lumps were visible. The reference for Acridine Orange was Vaseline (a petrolatum gel) mixed with the appropriate amounts of AO. The petrolatum gel was stirred for at least one hour and it was confirmed that no AO crystals were present prior to usage.

### Characterisation

#### Dispersed lipid phase

After processing, the dispersed lipid phase was investigated by means of light microscopy. The structures present are large (from a few microns up to hundreds of microns) and therefore the actual distribution was not considered to be crucial for penetration efficacy. No lumps were present in the formulation after processing. No difference between the formulations was observed regarding the dispersed lipid phase.

#### Ultra-fine dispersed phase

The properties of the small vesicle carriers were expected to have impact on the penetration profile. Therefore focus was set on characterisation of the vesicles in the ultra-fine dispersed phase. An autosizer (Malvern Zetasizer 1000HS, Malvern Instruments Ltd, Malvern, UK) was used to study the size distribution of the vesicles in the various vesicle fractions and a zetasizer (Malvern Zetasizer 2000, Malvern Instruments Ltd, Malvern, UK) was used to study the zeta potential. For proper determination dilution was necessary. The dilution may to a small extent result in reduced aggregation but is not expected to reduce the size of the primary structures (the vesicles). The adjuvants (dodecyl ethers) are expected to be more depleted from the membrane upon dilution but not to any extent that dramatically effects the size distribution. For the size distribution water was used and for the zeta-potential measurements 10 mM NaCl was used. The investigated parameters are shown in Table I and II in the Results section. For the particle diameter the presented value is an average of three averages obtained from ten single runs. The error presented is the standard error of the means. The zeta-potential is an average of five single runs. The error presented is the standard deviation.

**Table 1:** Characterisation data of the ultra-fine phase for 5 (6)-Carboxyfluorescein

	**CF-A**	**CF-B**	**CF-C**
**Particle diameter/nm**	276 ± 2	494 ± 20	975 ± 196
**Zeta-potential/mV**	-39.5 ± 0.5	-41.1 ± 0.7	-33.0 ± 2.4

**Table 2:** Characterisation data of the vesicle phase for Acridine orange 10-nonyl bromide

	**AO-A**	**AO-B**	**AO-C**
**Particle diameter/nm**	344 ± 8	1730 ± 994	1395 ± 194
**Zeta-potential/mV**	-36.0 ± 0.8	-35.3 ± 0.9	-30.5 ± 0.9

### Penetration in vitro model

#### Diffusion cell

Skin was mounted on diffusion cells (Laboratory Glass Apparatus Inc., CA, USA), with a surface area of 0.67 cm^2 ^and a receiver capacity of 3 ml, *stratum corneum *facing upwards.

Pig ear skin from pigham pigs, 7–8 months old, was used. The ears were obtained from Swedish Meat (Uppsala, Sweden) approximately two hours after slaughter and prepared the same day. Skin was removed with a scalpel. The appropriate thickness (640 μm) was obtained by use of a manual dermatome (Padgett Dermatome, Padgett Instruments Inc., Kansas City). An amount of 100 μl of the formulation was applied to the skin and carefully spread to cover the entire surface area. The formulations were applied within a week from the day of formulation. Because of the high viscosity of the formulations the exact amount was determined by weighing after application. For each formulation three experiments were performed, using skin patches from different animals. The receiver compartment was continuously rinsed with 25 mM HEPES buffer containing 133 mM NaCl (isotonic conditions). The pH of the buffer was set to 7.4 by addition of 1 M NaOH and before use the buffer was placed in an ultrasound bath for a few minutes to remove air bubbles. When connecting the buffer solution to the receiver compartment care was exercised to ensure that no air bubbles appeared on the dermal side of the skin or elsewhere in the receiver compartment. The receiver solution was continuously stirred using a small magnet and the temperature was maintained at 37°C throughout the experiment by coupling a water bath (HETO, Denmark) to the cells. The experiments were carried out for 24 hours under non-occluded conditions and fractions were collected from the receiver. The flow rate of the receiver solution was set to 1–2 ml/h and fractions were collected every 90 minutes throughout the experiment. After 24 hours the flow through the receiver compartment was stopped and the donor compartment was rinsed. First rinsing was performed with 10 ml HEPES buffer followed by 20 ml methanol. In cases where the formulation contained 10-nonyl bromide acridine orange the first rinsing step was excluded since the probe is not soluble in HEPES buffer.

#### Tape stripping procedure

The skin patch was put up on a board, the remaining dried formulation was removed and placed in HEPES buffer and the skin was stripped. The stripping was carried out using adhesive tape (Scotch magic tape, 3 M) by covering the area of the skin that had been in contact with the formulation with a piece of tape, 1.9 cm wide and 4 cm long. 15 strippings were performed to ensure that the *stratum corneum *was removed [15]. The first two tape strips were used to get rid of excess formulation on the skin. Strip 3–5 represent the upper *stratum corneum*, tape strip 6–10 represent the middle stratum corneum and the final 5 represent the lower *stratum corneum*. The strips were placed into 5 ml HEPES buffer or 5 ml methanol depending on the solubility of the probe. The remaining skin was placed in HEPES buffer for at least 48 hours and after that in methanol for at least 24 hours. These solutions are referred to as the extraction fractions. In cases where the formulation contained acridine orange 10-nonyl bromide the skin was only placed in methanol for at least 24 hours.

#### Fraction analysis

**Figure 1:**
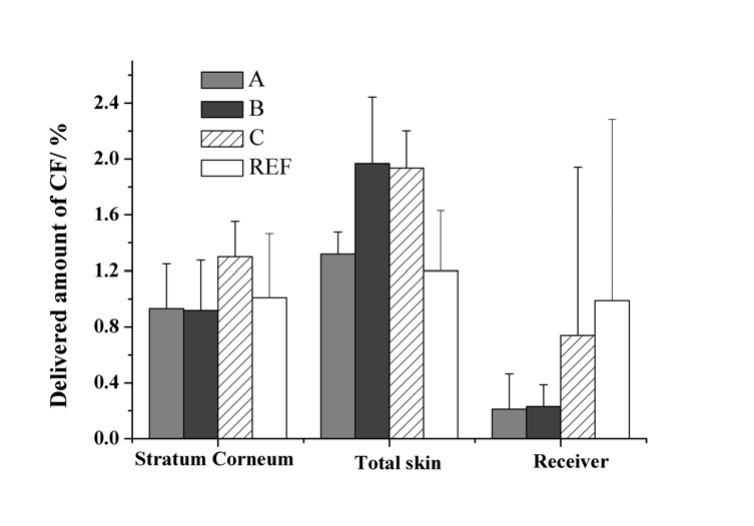
Amount of CF detected in stratum corneum, total skin and the receiver compartment. Errors are standard errors of mean.

**Figure 2:**
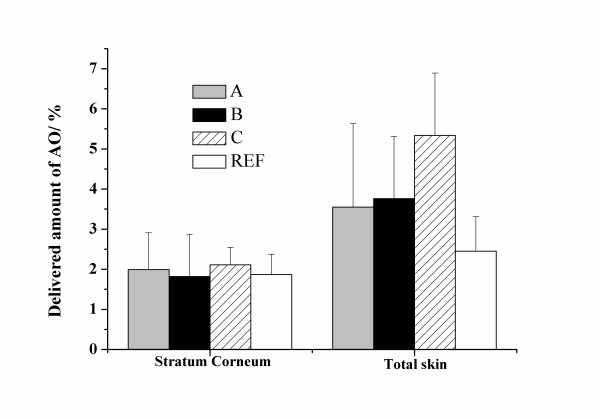
Amount of AO detected in stratum corneum and total skin. Errors are standard errors of mean.

**Figure 3:**
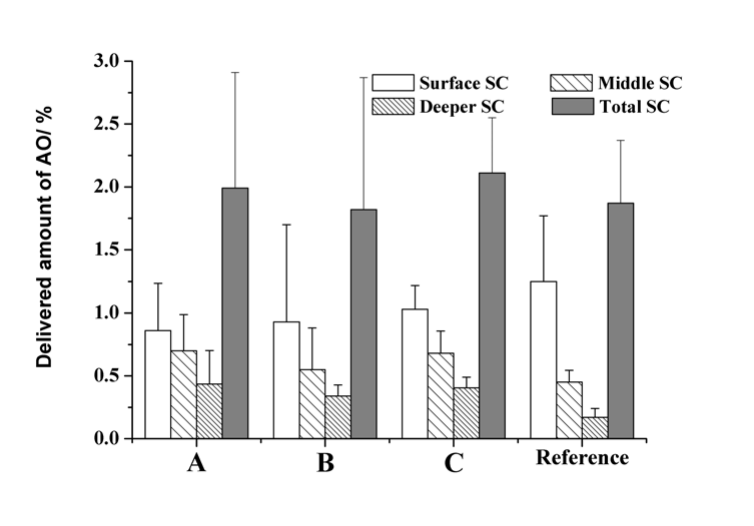
Amount of AO detected at different depth of stratum corneum. Errors are standard errors of mean.

The collected fractions were analysed by use of a spectrofluorimeter (FluoroMax-2, Instruments S.A, Inc.). The wavelengths of analysis were 490 nm (excitation) and 513 nm (emission) for CF in HEPES and 490 nm (excitation) and 517 nm (emission) for AO in methanol. Special care was taken in order for the fractions containing CF to be in a linear concentration to intensity region [16]. At the used wavelengths no interference of naturally occurring fluorescent components from skin could be detected. The data presented in Figures 1, 2, 3 are from three different runs for each formulation and each sample was analysed in triplicate. The errors shown are the standard errors of the means.

### In vivo penetration experiments

Tracer amounts of the fluorescent dye NDB C6-ceramide were added to the total lipid formulation and to the control (petrolatum). The total lipid formulation or control was applied to hairless mice epidermis. Skin biopsies, from three animals in each experimental group were taken two hours after application for fluorescence microscopy (Zeiss Axioplan 2).

## Results and discussion

### Skin penetration in vitro

The penetration performances of three different variations of the formulation were investigated by use of a pig ear skin diffusion cell. The variations were:

A) Formulation with 50% model active in dispersed lipid phase and 50% in ultra-fine dispersed lipid phase.

B) Formulation with all model active in untra-fine dispersed lipid phase.

C) Formulation containing the non-ionic adjuvants, tetraethylene glycol monododecyl ether and polyoxyethylene 23 glycol monododecyl ether, with 50% model active in dispersed lipid phase and 50% in ultra-fine dispersed lipid phase.

The penetration of the formulations were compared with appropriate references. Pigskin was chosen since its stratum corneum structure resembles human stratum corneum more than does mouse skin [17].

Firstly, the permeation of the hydrophilic CF was investigated. The results for CF (Fig. 1) show the amount detected in the stratum corneum, the total amount detected in all skin fractions and the amount detected in the receiver.

The amount detected in stratum corneum is similar for all four formulations except for CF C that contains polyoxyethylene alkyl ethers. The non-ionic surfactants are expected to work in a similar fashion as do other non-ionic surfactants, such as Tween 20 or Synperonics [7]. The larger amount found in the stratum corneum for CF C shows that the adjuvants are successful. This is emphasised by the fact that even though the total amount delivered to skin is similar for CF-B and CF-C the amount found in the receiver is much larger for CF-C. Interstingly, there is also a clear difference between CF-A and CF-B when it comes to overall delivery to the skin. This shows that it is beneficial to distribute more of the water soluble CF in the ultra-fine dispersed phase with vesicles when a deeper delivery is aimed at. Importantly, this also shows that there is an effect of the vesicle carrier as such and that the penetration improvement does not come from the lipid matrix functioning as a penetration enhancer alone. The effect is probably due to an ability of small vesicles to function as carriers down to a certain depth of the skin. The characteristics of the vesicles in the ultra-fine dispersed phases are shown in Table 1. The somewhat larger average size obtained for CF-B compared to CF-A is believed to be process dependent. The close to micron size for CF-C on the other hand is most likely due to interactions between the adjuvants and the lipid material. The data show that the presence of the non-ionic adjuvants, tetraethylene glycol monododecyl ether and polyoxyethylene 23 glycol monododecyl ether (formulations C), results in larger vesicle sizes and a somewhat smaller magnitude of zeta-potential. In this context it is important to realise that inclusion of these surfactants in the vesicle membrane results in ethylene oxide coils reaching out from the vesicle surfaces. A reason for the increase in size may well be that a lateral pressure between ethylene oxide coils in the membrane (especially on the inside) counteracts creation of very small vesicles. The present coils also reduce mobility of the vesicles but due to the low polymer density they are not expected to contribute much to the scattering pattern. This results in a decrease in measured zeta-potential and has previously been seen for similar systems [18]. Here it is worth pointing out that the mechanism of drug carrier penetration in general is more complex than just a matter of size. This is manifested in findings that drugs solubilised in micelles have had worse penetration abilities than corresponding drugs solubilised in vesicles [19].

Another interesting feature is that the largest amount of active found in the receiver was for the reference xanthan gum formulation. Thus the delivered active is not retained but more easily transferred to the receiver than is the active when formulated in the lipid matrix gel. The behaviour of the reference formulation deserves some comment. The explanation for it being so efficient is probably that the amount of free water is larger in the reference and that water is known to work as a penetration enhancer [7]. Moreover the conditions with a large amount of free water in a gel layer of non-negligable height are close to occluded conditions. Such conditions have been reported beneficial for uptake of drugs [20,21]. In contrast, the slow release from the lipid formulations to the receiver may in fact indicate a slow release mechanism that is beneficial for many topical treatments since systemic toxicity is more easily avoided.

In addition, the penetration behaviour of the lipohilic molecule AO was investigated. The results for the different formulations are shown in Figure 2. No amount of AO could be detected in the receiver. This is not surprising since the solubility in water is low.

Similar to the formulations containing CF the amount in total skin is highest for the formulation containing the non-ionic adjuvants. Thus, these adjuvants work well both for hydrophilic and lipophilic substances. Interestingly, the distribution of all AO into the vesicle phase (AO-B) did not have any beneficial effect on the penetration. This may, in part, be due to the fact that the homogenisation was less efficient in the presence of all AO. This is manifested in that the obtained vesicle size was large (Table II) and is probably due to that the lipophilic probe interacts with the membrane in destabilising manner. Even though one could see a small tendency also for CF (CF-B) we do not believe that CF interacts with the membrane to any large extent.

Importantly, all lipid matrix formulations were superior to the reference (vaseline) as far as total delivery of AO to skin is concerned. In Figure 2 there is no difference in delivery to stratum corneum. If a closer look is taken at three different depth of stratum corneum, however, differences can be seen (Figure 3). From the data it is evident that for formulations A, B and C the deeper layers contain more AO than does the reference. In conclusion, the formulation containing adjuvants (C) delivered more than twice the amount delivered by the reference overall (see total skin in Fig. 2) which is considered a significant improvement. If the deeper parts of the skin (total skin excluding SC) is considered the increase in delivery is over four times better.

### In vivo penetration experiments

The lipid formulations were thus successful concerning the delivery aspects as determined by the in vitro test. For the obtained data to be even more sensational they should be qualitatively applicable to in vivo situations. Thus, an in vivo model was set up in order to establish the improved performance over reference for a lipophilic substance.

**Figure 4:**
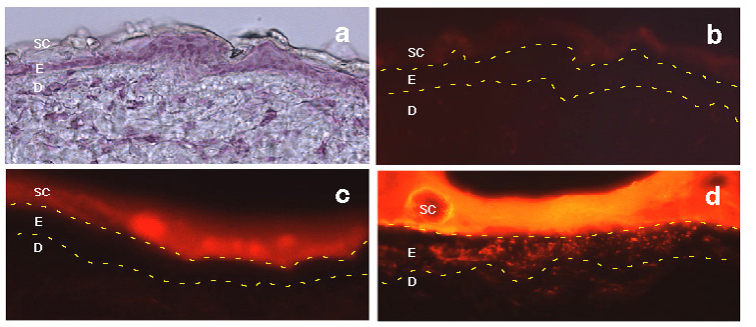
*Fluorescence microscopy images*. a) frozen section of normal skin stained with hematoxylin viewed by light microscopy, b) auto fluorescence of untreated epidermis, c) auto fluorescence of petrolatum with penetration only into stratum corneum, d)auto fluorescence of the total lipid formulation (variation A) with penetration into the viable epidermis and dermis.

The fluorescent microscope image (Figure 4) clearly shows that the uptake of the fluorescent probe is more enhanced for the lipid matrix formulation than for the reference. Remarkably, the effect is seen already 2 hours after application. It is thus plausible that the in vitro data presented above correspond to the same trends in vivo even though exact behaviour is expected to depend on many factors such as species, age and individual variations of other kinds.

## Conclusion

A new water-based lipid formulation is presented that provides good penetration properties for both lipophilic and hydrophilic drugs. The composition of the formulation was chosen with the aim of a small a disturbance of the skin barrier function as possible. This effect will be addressed in coming studies.

The formulation showed delivery properties exceeding that of the references in the pigskin *in vitro *model. The effect is largest for lipohilic AO (Acridine orange 10-nonyl bromide) where all lipid matrix formulations were superior in amount detected in the skin. The results for the hydrophilic CF (5(6)-carboxyfluorescein) were also promising. Especially efficient was the lipid formulation containing the non-ionic adjuvants tetraethylene glycol monododecyl ether and polyoxyethylene 23 dodecyl ether.

The additional *in vivo *study suggests that the used *in vitro *model has qualitative bearing on relevant *in vivo *situations.

## Abbreviations

CF: 5(6)-carboxyfluorescein, AO: Acridine orange 10-nonyl bromide
